# Diagnostic Performance and Cell Count of EBUS–TBNA Needle Gauges: A Prospective Trial

**DOI:** 10.3390/jcm12124033

**Published:** 2023-06-13

**Authors:** Juliana Guarize, Cristina Diotti, Monica Casiraghi, Stefano Donghi, Clementina Di Tonno, Patrizia Mancuso, Laura Zorzino, Giulia Sedda, Davide Radice, Luca Bertolaccini, Lorenzo Spaggiari

**Affiliations:** 1 Interventional Pulmonology Unit, IEO, European Institute of Oncology IRCCS, 20141 Milan, Italy; juliana.guarize@ieo.it (J.G.); stefano.donghi@ieo.it (S.D.); 2 Department of Thoracic Surgery, IEO, European Institute of Oncology IRCCS, Via Ripamonti 435, 20141 Milan, Italy; cristina.diotti@ieo.it (C.D.); monica.casiraghi@ieo.it (M.C.); giulia.sedda@ieo.it (G.S.); lorenzo.spaggiari@ieo.it (L.S.); 3 Department of Oncology and Hemato-Oncology, University of Milan, 20122 Milan, Italy; 4 Division of Pathology, IEO, European Institute of Oncology IRCCS, 20141 Milan, Italy; clementina.ditonno@ieo.it; 5 Division of Clinical Hemato-Oncology Laboratory, IEO, European Institute of Oncology IRCCS, 20141 Milan, Italy; patrizia.mancuso@ieo.it; 6 Division of Laboratory Medicine, IEO, European Institute of Oncology IRCCS, 20141 Milan, Italy; laura.zorzino@ieo.it; 7 Division of Epidemiology and Biostatistics, IEO, European Institute of Oncology IRCCS, 20141 Milan, Italy; davide.radice@ieo.it

**Keywords:** endobronchial ultrasound-GUIDED transbronchial needle aspiration, 19-G flex needle, diagnostic yield, cell count, lung cancer

## Abstract

Background. Endobronchial ultrasound-guided transbronchial needle aspiration (EBUS-TBNA) is a well-established diagnostic procedure for evaluating hilar and mediastinal lymphadenopathies and is the gold standard for lung cancer diagnosis and staging. Recent studies assessed the effectiveness of the 19-G flex needle in obtaining larger EBUS-TBNA samples, and prospective small series gave similar results in terms of diagnostic yield when testing different gauge needles. The lack of homogeneity between series and the small sample size of some prospective cohorts poses a limit to the validity of those results. This prospective controlled study compared the 19-G flex and 22-G needles in terms of diagnostic yield. An objective laboratory method was used to count cells and compare the two needles’ cytologic yields. Material. A prospective controlled study was conducted on 90 patients undergoing EBUS-TBNA for the diagnosis of hilar and mediastinal lymphadenopathies. The institutional ethic committee (IEO573) approved the study, and informed consent was obtained from all patients. Results. A total of 90 patients were enrolled in this study, 84.4% of whom were diagnosed with malignancy and 15.6% with non-neoplastic disease. Sensitivity for malignancy was 93.4% (CI: 87.4–97.1%) for the 19-G needle and 92.6% (CI: 86.3–96.5%) for the 22-G needle (*p* = 0.80). The percentage of malignant cells in the cell block was 63.9% and 61.5% for the 22-G and 19-G needles, respectively. The cell count assessed by flow cytometry was 2071 cells/µL (IQR: 600,2265) with the 22-G needle and 2761 cells/µL (IQR: 505,3250) with the 19-G needle (*p* = 0.79). The malignant cell count was 0.05 × 10^3^ cells/µL with the 22-G and 0.08 × 10^3^ cells/µL with the 19-G needle (*p* = 0.70). There was no difference in the presence of tissue cores in the samples, and rapid on-site evaluation (ROSE) cellularity was comparable between the two needles. Conclusions. The 19-G flex EBUS-TBNA needle is comparable to the 22-G needle in terms of diagnostic yield for cyto-histological evaluation of hilar and mediastinal lymphadenopathies. There is no difference between the 19-G and 22-G needle cell counts evaluated by flow cytometry.

## 1. Introduction

Endobronchial ultrasound transbronchial needle aspiration (EBUS-TBNA) emerged as a minimally invasive procedure able to provide lymph node diagnosis and staging in mediastinal and hilar lymphadenopathies with high accuracy in different clinical scenarios [1,2].

EBUS-TBNA currently represents the gold standard for pathological characterization and mediastinal staging in lung cancer patients [3].

Molecular targeted therapies have radically changed the management of lung cancer and made accurate histological and cytological sampling for the assessment of programmed death-ligand 1 (PD-L1) expression mandatory. EBUS-TBNA is the procedure of choice for molecular testing in advanced-stage lung cancer patients [4,5].

As new EBUS-TBNA devices are developed, the need for larger needle sizes to provide larger samples remains a significant matter of debate.

Retrospective series addressed the possibility of obtaining larger tissue samples with the 19-G flex needle, thus theoretically increasing EBUS-TBNA diagnostic performance despite the much higher cost of the 19-G needle [6,7,8].

Prospective series obtained equivalent diagnostic yields with different needle gauges [9,10]. However, the actual ability of the 19-G flex needle to provide larger tissue samples and the number of cells obtained when sampling with different needle gauges have never been addressed. 

This prospective study was designed to assess if, when compared to the 22-G needle, the 19-G flex needle is able to provide samples with higher cellularity as assessed in cell block slices and by flow cytometry.

## 2. Material and Methods

A prospective controlled study was conducted on 90 patients undergoing EBUS-TBNA for the diagnosis of hilar and mediastinal lymphadenopathies. The institutional ethic committee (IEO573) approved the study, and informed consent was obtained from all patients.

Before EBUS-TBNA procedures, all patients underwent noninvasive evaluation with computed tomography (CT) and positron emission tomography with 18fluorodeoxyglucose (PET-FDG) scans. A lymph node was considered clinically suspect if greater than 10 mm in the short axis on a CT scan and with increased pathological uptake on a PET-FDG scan.

Patients underwent bronchoscopy under moderate sedation and spontaneous ventilation. Two experienced interventional pulmonologists performed EBUS-TBNA.

Lymph node ultrasound characteristics were recorded, including shape, margins, echogenicity, central hilar structure, and coagulation necrosis signs. 

All lymph nodes were sampled with both the 22-G and the 19-G needle. Three needle passages were performed with each needle for each lymph node, and one additional passage was performed for cell count evaluation. 

A small amount of needle sample was smeared onto a glass slide for rapid on-site evaluation (ROSE), and the number of passages performed to achieve specimen adequacy was recorded for each needle. 

Specimens were evaluated using direct visual microscopic inspection (criteria for specimens’ evaluation are described above), and flow cytometry was used to assess the number of cells obtained with each needle objectively. 

Lymph nodes negative for malignancy and without a specific non-neoplastic diagnosis at EBUS-TBNA underwent mediastinoscopy and/or video-thoracoscopy for a definitive surgical diagnosis.

The objective of the study was to compare the 19-G Flex and 22-G EBUS-TBNA needles in terms of both diagnostic and cytological yields, the latter being evaluated using both cell-block pathological specimens and flow cytometry assay. 

ROSE adequacy, presence of tissue core, and necrosis with both needles were also assessed. 

### 2.1. Specimen Processing and Evaluation Criteria

For the EBUS-TBNA specimens, each needle was centrifuged for 7 min, covered with agarose to obtain a cell block, then fixed in formalin and stained with hematoxylin and eosin stain. A single cytopathologist evaluated cell blocks.

### 2.2. Cell Block Evaluation Criteria on Hematoxylin and Eosin-Stained Sections

- Overall cellularity: overall cellularity visible in 20× magnification microscopic fields (excluding red blood cells) and classified as low (present in less than 30% of the slices), moderate (present in 30–60% of the slices), or high (present in more than 60% of the slices).
- Percentage of neoplastic cells: number of neoplastic cells present on the slices evaluated on the HE slices at 20× magnification microscopic fields.
- The percentage of nonmalignant cells is the number of lymph node cells evaluated on 20× magnification microscopic fields.
- Necrosis: classified as present or absent.
- Tissue core: proportion of cells visible as tissue cores, excluding red blood cells, evaluated at 20× magnification microscopic fields and classified as low (present in less than 30% of the slices), moderate (present in 30–60% of the slices), or high (present in more than 60% of the slices).
- ROSE evaluation criteria in May–Grunwald–Giemsa-stained sections:
- A cytopathologist performed ROSE. Smears were evaluated for overall cellularity, tumor cells, and blood.
- Overall cellularity: comprehensive cellularity evaluated on 4× magnification microscopic fields, excluding red blood cells and classified with categorical variables as low (present in less than 30% of the slices), moderate (present in 30–60% of the slices), or high (present in more than 60% of the slices).
- Percentage of neoplastic cells: number of neoplastic cells evaluated on 4X magnification microscopic fields.
- The percentage of nonmalignant cellularity is the number of lymph node cells evaluated on 4× magnification microscopic fields.

### 2.3. Cell Count Processing by Flow Cytometer

For each lymph node sampled and for each needle, one needle passage was placed into a saline solution for processing and cell count in the laboratory. 

Cell count was performed using the channel dedicated to the analysis of biological fluids in the automated blood cell counter XN 2000 (Sysmex Corporation, Kobe, Japan). As this instrument automatically verifies the background and the extension of the cell count, it allows automated analysis without samples’ pretreatment, and this ensures more excellent reproducibility and precision of the results even in samples containing few cells.

The count of nucleated cells (leukocyte and nonleukocyte) was carried out using the principle of fluorescence flow cytometry, where the intensity of the fluorescence signal is directly proportional to the content of nucleic acids. The number of total leukocytes and non-nucleated cells (TC-BF 10^3^/µL), the number of white blood cells (WBC-BF 10^3^/µL), and the number and percentage of highly fluorescent cells (tumor cells HF 10^3^/µL, HF%) were all evaluated.

For neoplastic cell marking, samples were incubated for at least 20 min at 4 °C with the DNA-binding antibodies anti-7-amino actinomycin (7AAD) and Syto16 (to exclude apoptotic cells, both Thermo Fisher Scientific, Eisai, Medipost, USA) together with anti-CD45 lymphocyte marker (to exclude hematopoietic cells; TF) and anti-CD326 (epithelial cell adhesion molecule or EpCAM). Flow cytometry was analyzed using the FACS Canto instrument (Becton Dickinson). For nonmalignant samples, viable cells were incubated with different lymphocyte markers for at least 20 min at 4 °C: CD3+/CD4+ and CD3+/CD8+ for T lymphocytes, CD19+ for B cells, CD3+/CD56+ for NK lymphocytes, and CD45+ to exclude hematopoietic cells (all TFs). After incubation, samples were acquired using a 3-laser–10-color flow cytometer (Navios, Beckman Coulter, Brea, CA, USA). Cells were counted for each of the two needles, and global cellularity and specific count for tumor cells were determined.

### 2.4. Statistical Analysis

The sample size was calculated in order to detect a minimum of 15% difference between the two needles with regards to the quality of the diagnostic tissue (obtained by three punctures per lymph node), using a two-sided binomial test. Setting the power to 80% at the 5% significance level, the minimum number of patients required was N = 90. Categorical and continuous variables for patients, as well as for lymph nodes, were summarized either by count and percent (categorical) or mean (continuous) and either standard deviation (SD) or interquartile range (IQR), respectively, for skewed distributions. Diagnostic accuracy measures were tabulated alongside 95% confidence intervals (95% CI). Agreement between EBUS-TBNA 19-G and 22-G was tested using Cohen’s kappa coefficient. The significance of each EBUS-TBNA 19-G vs. 22-G comparison was assessed using the McNemar test for categorical variables with 2 × 2 tables and the Bowker symmetry test for variables with more than two levels. The signed-rank sum test was used to compare continuous variables. Bland and Altman plots for laboratory data were produced as well. The normality assumption for continuous variables was tested using the Shapiro–Wilk test. All tests were two-tailed and considered significant at the 5% level. All analyses used SAS 9.4 (Cary, NC, USA).

## 3. Results

A total of 90 patients were enrolled in the study. Demographic characteristics included 36 men with an average baseline age of 65 years. Patient demographics are reported in Table 1. 

A total of 121 lymph nodes (LN) were sampled; the average size of LN was 14 mm. One LN station was sampled in 65 patients, two LN stations in 22 patients, and three LN stations in 25 patients. The most frequently sampled stations were 4R and 7, followed by 11L. The lymph node station characteristics are shown in Table 2.

EBUS-TBNA diagnosis included malignancy in 84.4% (55.6% NSCLC, 4.4% SCLC, 23.3% metastases of previous malignancies, 1.1% Hodgkin Lymphoma) and non-neoplastic disease in 15.5% of cases (11.1% granulomatous inflammation and 4.4% reactive lymphadenopathy). In 17 cases, the 22-G needle demonstrated reactive lymphadenopathy, and in 3 of them, the 19-G needle provided a definitive diagnosis of granulomatous inflammation. EBUS-TBNA diagnoses are summarized in Table 3. 

Of the 15 patients with a nonspecific diagnosis of EBUS-TBNA, 3 declined mediastinoscopy and were included in false negative cases. 

Overall sensitivity for malignancy was 92.6% (95% CI: 86.3–96.5%) for the 22-G needle and 93.4% (95% CI: 87.4–97.1%) for 19-G (*p* = 0.80). The false negative rate was 7.4% (3.5–13.6%) for 22-G and 6.6% (2.9–12.6%) for 19-G (*p* = 0.80), with no difference between the two needles (Table 4).

The cell block cellularity was “high” in 54 (44.6%) with 22-G and 62 (51.2%) lymph nodes with 19-G needle, and ROSE cellularity was “high” in 51 (42.2%) and 64 (52.9%) with 22-G and 19-G needles respectively, with no statistical difference (Table 5). 

**Figure 1:**
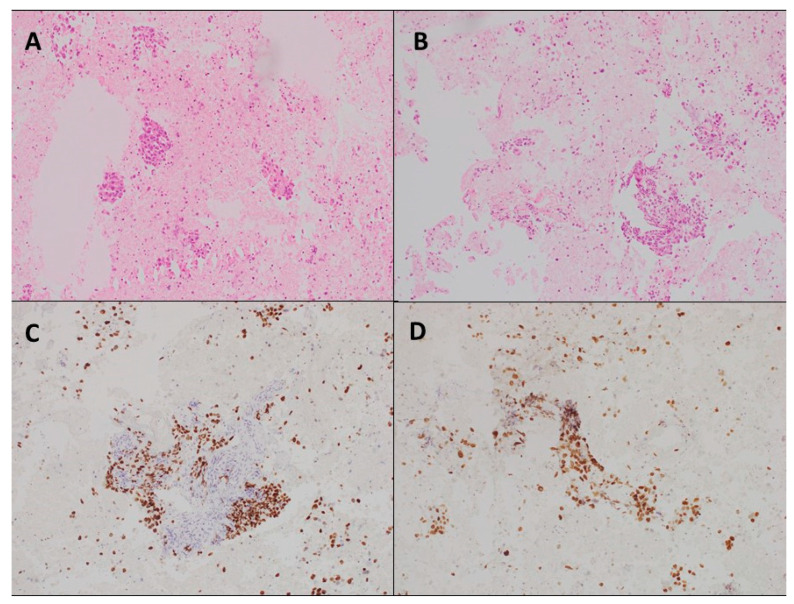
Specimens of lymph-node metastasis of lung adenocarcinoma. (**A**) 22-G needle and (**B**) 19-G needle hematoxylin–eosin stain, 10× magnification; (**C**) 22-G needle and (**D**) 19-G needle immunocytochemistry showing the TTF1 positive cells, 10× magnification.

The percentage of malignant cells in the cell block was similar between the two needles (63.9% for the 22-G and 61.5% for the 19-G) (*p* = 0.29). Figure 1 shows cell block slices for the two needles.

One needle passage was sufficient to obtain sample adequacy in 106 (88%) and 102 (84%) lymph nodes for the 19-G and 22-G, respectively, with no statistical differences (*p* = 0.45). For one lymph node, adequacy was not achieved after three needle passages at ROSE, and all needle passages were processed in the cell block evaluation (Table 6).

The presence of necrosis was observed in 38 (31.4%) samples, with a similar distribution between the two needles (*p* = 0.32). 

Tissue cores were present in 41 samples. A small amount of tissue core (<30%) was observed in 8 (19.5%) 22-G samples and 5 (12.2%) 19-G samples; amounts of tissue core between 30 and 60% were found in 15 (36.6%) cases for each needle, and high amounts of tissue core (>60%) were observed in 18 (43.9%) cases with 22-G and 21 (51.2%) cases with 19-G, with no statistical difference (*p* = 0.61) (Table 7).

Flow cytometry cell count showed no difference in overall cellularity between the two needles: 2071 cells/µL (IQR: 600,2265) with the 22-G needle and 2761 cells/µL (IQR: 505,3250) with the 19-G needle (*p* = 0.79). 

The malignant cell count (0.05 × 103 cells/µL for the 22-G and 0.08 × 103 cells/µL for the 19-G) was similar between the two needles. 

**Figure 2:**
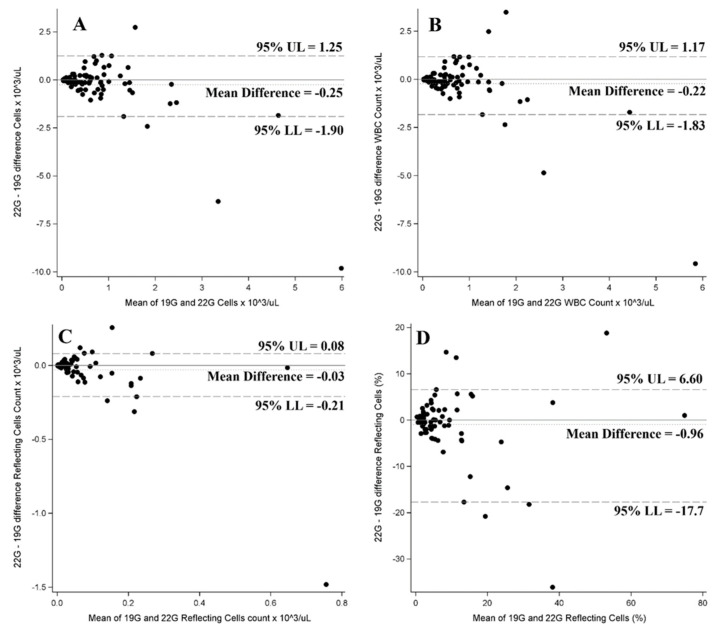
Bland–Altman plots for the comparison of 22-G vs. 19-G. (**A**) Cells; (**B**) WBC; (**C**) reflecting cells count; (**D**) reflecting cells.

The flow cytometry assay observed no difference between the two needles in cell type distribution. Summary statistics for flow cytometry data for both needles are shown in Table 8. Flow cytometry results for the two needles are compared in Figure 2. Complications occurred in five cases (5.6%): three self-limited bleeding after 19-G needle sampling and two cases of intraprocedural desaturation.

## 4. Discussion

EBUS-TBNA is the procedure of choice for invasive mediastinal staging and diagnostic tissue sampling in thoracic diseases [1,11]. EBUS-TBNA is often employed to stage advanced lung cancer as it is a minimally invasive procedure able to provide samples suitable for the nowadays mandatory molecular analysis, NGS sequencing, and PD-L1 assessment [5,6]. The need for large tissue samples is still a topic of debate, especially when multiple gene analysis is required, and other thoracic diseases such as granulomatous inflammation and lymphoproliferative disorders should also be considered. In clinical practice, most EBUS-TBNA procedures still employ the 22-G needle, although recent studies have suggested that the 19-G flex needle could provide higher amounts of tissue samples for cell block specimens and molecular analysis with better diagnostic yield despite the elevated costs [7]. Some prospective series suggested similar diagnostic yields for different EBUS-TBNA needle gauges without explicitly evaluating the cellularity effectively sampled by the different needles [12,13].

The usefulness of the flex 19-G needle in mediastinal lymph nodes assessment was retrospectively evaluated in a small cohort study which concluded that the 19-G needle has the potential to provide larger volumetric and core tissue samples and can be considered in selected cases where more significant tissue acquisition is required, such as for molecular analyses and PD-L1 expression testing [7]. Another prospective pilot study evaluated the 19-G needle performance in the diagnosis of lung cancer and molecular markers assays, concluding that the 19-G needle has a high rate of success [12]. In a small prospective cohort, an examination of the cellular material in the cell blocks obtained with the 19-G and 21-G needles concluded that the cell area was significantly large with the 19-G needle [13]. Alternatively, in a prospective study comparing the diagnostic yield of 19-G and 21-G needles, the authors reported no significant difference between them and suggested a higher diagnostic yield in cancer lymph nodes with the 19-G needle [10]. A recent randomized trial included a small cohort of 40 patients (56 lesions sampled) for the evaluation of the diagnostic yield of the 22-G and the 19-G needles in lymph nodes and lung masses. The authors reported no significant difference between the two needles in terms of diagnostic yield and molecular testing [14]. This prospective study compared 19-G flex and 22-G EBUS-TBNA needle samples by looking at their diagnostic yields in pathological analysis and objectively assessing their overall cellularity and malignant cell count. Ninety patients were included, and 121 lymph node stations were sampled with both needles. The same number of passes was performed with each needle in all stations, avoiding collection sample bias and allowing a precise comparison between the two needles. The results of our study show no difference in diagnostic yield and similar sensitivity for malignant and nonmalignant diseases between the two needles (92.6% (95% CI: 86.3–96.5%) for 22-G needle and 93.4% (95% CI: 87.4–97.1%) for 19-G (*p* = 0.80)). Similar false negative rates were observed with the two needles (7.4% (3.5–13.6%) for 22-G and 6.6% (2.9–12.6%) for 19-G (*p* = 0.80)). The presence of tissue cores and the cellularity observed in the cell blocks were similar between the two needles (high cell block cellularity was observed in 54 (44.6%), and 62 (51.2%) cases and high amounts of tissue cores were observed in 18 (43.9%) and 21 (51.2%) cases with the 22-G and 19-G needle, respectively). 

ROSE evaluation showed no difference between the two needles in terms of both cellularity and the number of needle passes performed to obtain adequacy during the procedure. Flow cytometry showed no difference in cell count between the 22-G and the 19-G needles samples: 2071 cells/µL (IQR: 600,2265) with the 22-G, and 2761 cells/µL (IQR: 505,3250) with the 19-G (*p* = 0.79). Malignant cell count was also comparable between the two needles (0.05 × 103 cells/µL for the 22-G and 0.08 × 103 cells/µL for the 19-G). There was no difference in terms of diagnostic yield, sensitivity for malignancy, presence of tissue cores, and cell count obtained with the two needles addressed by flow cytometry.

Our study presents some limitations: the lack of randomization and blinding, adding potential selection and performance biases, and the potential impact of multiple needle passes on sample quality. Future research should seek to solve these constraints by integrating randomization and evaluating the possible impact of different sample quality passes. 

## 5. Conclusions

The current study is the largest prospective cohort study, comparing the 19-G and 22-G needles by objectively evaluating the cytological yield in EBUS-TBNA samples with a laboratory cell counting method. 

Based on our findings, EBUS-TBNA procedures performed by experienced physicians do not benefit from the use of larger needles.

## Figures and Tables

**Table 1:** Patients’ characteristics, N = 90.

Characteristics		Statistics ^1^
Age, years		65.1 (9.0)
Female Sex		44 (48.9)
Comorbidities ^2^	Heart	47 (52.2)
	Lung	14 (15.6)
	Diabetes	7 (7.8)
	Other	18 (20.0)
	Total ^3^	71 (78.9)
Smoking status	Smoker	44 (48.9)
	Never or Former	46 (51.1)
ASA	1	4 (4.4)
	2	73 (81.1)
	3	13 (14.4)
Lung nodules, N = 76	Single	45 (59.2)
	Multiple	31 (40.8)
Procedure duration (min)		38.5 (13.9)
Procedure complications		5 (5.6)

^1^ Statistics are as follows: mean (SD) for continuous variables, N (%) on non-missing cases otherwise. ^2^ Non-mutually exclusive. ^3^ Number of patients with at least one comorbidity.

**Table 2:** Frequency distribution and summary statistics of lymph node dimension (mm) by the station at echography.

Lymph Node Station	N (%)	Mean (SD)
4R	47 (38.8)	12.0 (4.0)
7	37 (30.6)	17.4 (5.9)
11L	11 (9.1)	13.5 (2.8)
11R	8 (6.6)	15.7 (5.0)
4L	8 (6.6)	10.2 (3.4)
2R	3 (2.5)	9.9 (1.8)
ST1	3 (2.5)	13.9 (7.1)
10R	2 (1.7)	10.3 (0.2)
10L	1 (0.8)	24.2
2L	1 (0.8)	9.7
Overall	121 (100)	14.0 (5.3)

Significant pairwise comparisons: 4R vs. 7, *p* < 0.001; 4L vs. 7, *p* = 0.009. For all other comparisons, *p* > 0.08.

**Table 3:** Definitive diagnosis for N = 90 patients.

Diagnosis	N (%)
NSCLC	50 (55.6)
Metastasis from the previous tumor	21 (23.3)
Granulomatous inflammation	10 (11.1)
Reactive lymph nodes	4 (4.4)
SCLC	4 (4.4)
Classical Hodgkin lymphoma	1 (1.1)

**Table 4:** Diagnostic accuracy for N = 121 lymph nodes.

Measure	EBUS-TBNA	Estimate, % (95% CI)	*p*-Value
Sensitivity	19	93.4 (87.4, 97.1)	
	22	92.6 (86.3, 96.5)	0.80
FNR	19	6.6 (2.9, 12.6)	
	22	7.4 (3.5, 13.6)	0.80

FNR = false negative rate; 95% CI = 95% confidence interval; EBUS-TBNA 19-G and EBUS-TBNA 22-G agreement: kappa coefficient = 0.81, 95% CI: (0.60, 1.00), *p* = 0.56.

**Table 5:** EBUS-TBNA 22-G vs. 19-G cellularity comparisons N (%), N = 121.

	22-G	19-G		Total	*p*-Value ^a^
		Below Average/Average	Above Average		
ROSE	Low/Moderate	38 (31.4)	31 (26.5)	70 (57.9)	
	High	19 (15.7)	32 (26.5)	51 (42.2)	0.07
	Total	57 (47.1)	64 (52.9)	121 (100)	
Cell-block	Low/Moderate	40 (33.1)	27 (22.3)	67 (55.4)	
	High	19 (15.7)	35 (28.9)	54 (44.6)	0.24
	Total	59 (48.8)	62 (51.2)	121 (100)	

**^a^** McNemar’s test.

**Table 6:** Steps count comparison for sample adequacy, N (%).

22-G Steps Count	19-G Steps Count		Total	*p*-Value
	1	2–3		
1	90 (74.4)	12 (9.9)	102 (84.3)	
2–3	16 (13.2)	3 (2.5)	19 (15.7)	0.45
Total	106 (87.6)	15 (12.4)	121 (100)	

One lymph node subject did not obtain adequacy after 3 steps with EBUS-TBNA 22-G.

**Table 7:** Tissue core comparison, N (%).

22-G	19-G			Total	*p*-Value ^a^
	<30%	30–60%	>60%		
<30%	4 (5.8)	0	4 (9.8)	8 (19.5)	
30–60%	0	15 (36.6)	0	15 (36.6)	0.61
>60%	1 (2.4)	0	17 (41.5)	18 (43.9)	
Total	5 (12.2)	15 (36.6)	21 (51.2)	41 (100)	

^a^ Symmetry test.

**Table 8:** EBUS-TBNA 22-G vs. 19-G cytofluorimetry data comparisons.

Parameter	Needle	N	Mean (IQR)	*p*-Value ^a^
Cells Count (/uL)	22-G	120	2071 (600,2265)	
	19-G	120	2761 (505,3250)	0.79
Vitality (%)	22-G	120	56.3 (36,78)	
	19-G	120	60.1 (43,81)	0.21
Lymphocytes (%)	22-G	120	42.8 (12,70)	
	19-G	120	43.4 (14,67)	0.75
T4 cells (%)	22-G	18	45.2 (27,62)	
	19-G	18	51.2 (41,63)	0.77
T8 cells (%)	22-G	18	15.2 (10,16)	
	19-G	18	13.9 (11,17)	0.77
NK cells (%)	22-G	18	1.8 (1.0,2.0)	
	19-G	18	2.5 (1.0,2.0)	0.72
B cells (%)	22-G	18	18.1 (5,29)	
	19-G	18	17.4 (5,26)	0.87

**^a^** Signed-rank sum test; IQR = interquartile range.

## Data Availability

The data presented in this study are available on request from the corresponding author. The data are not publicly available due to IEO Policy.
